# Global trends, research hotspots, and clinical evidence mapping of music therapy interventions for cognitive impairment: a bibliometric and visualization analysis from 2006 to 2026

**DOI:** 10.3389/fpsyg.2026.1917320

**Published:** 2026-09-24

**Authors:** Qiangli Lei, Qing Liu

**Affiliations:** 1 College of Music, Hengyang Normal University, Hengyang, China; 2 School of Physical Education and Sports Science, South China Normal University, Guangzhou, China

**Keywords:** Alzheimer’s disease, bibliometrics, cognitive impairment, dementia, music therapy, randomized controlled trial

## Abstract

**Background:**

Cognitive impairment is an important public health issue in ageing societies. Music therapy, as a safe and accessible non-pharmacological intervention, has been increasingly used in the management of cognitive impairment. However, the overall research pattern, evolution of research hotspots, and features of clinical evidence in this field have not been systematically reviewed. This study aimed to analyze the current status, major topics, and development trends of research on music therapy interventions for cognitive impairment from 2006 to 2026.

**Methods:**

A stratified multi-database search strategy was used. The Web of Science Core Collection (WoSCC) served as the main data source. Bibliometric and visualization analyses were performed using Bibliometrix, CiteSpace, and VOSviewer. The Scopus database was also used to cross-validate key findings. In addition, randomized controlled trials (RCTs) retrieved from PubMed were analyzed to further assess the clinical evidence base.

**Results:**

A total of 835 publications were included from WoSCC. From 2006 to 2025, the annual number of publications increased steadily, with an average annual growth rate of 13.35%. The publication output reached its peak in 2025, and the exponential model showed a good fit to the growth trend (*R*^2^ = 0.913). The United States, China, the United Kingdom, and Australia were the leading contributing countries, while institutions such as the University of Melbourne made notable contributions. The main research topics focused on dementia, music therapy, Alzheimer’s disease, quality of life, and cognitive dysfunction. Over time, research hotspots have extended toward cognitive improvement, long-term care, and neuroplasticity-related mechanisms. A total of 250 RCTs were identified from PubMed, indicating a growing body of clinical evidence.

**Conclusion:**

Research on music therapy interventions for cognitive impairment has shown continuous growth. Core topics mainly involve cognitive function, behavioral symptoms, quality of life, and care support. Mechanistic studies, especially those related to neuroplasticity, have become an important research frontier. Future studies should place greater emphasis on high-quality clinical research, clarify the mechanisms of music therapy, and promote its precise application and clinical translation.

## 1 Introduction

Cognitive impairment is an important public health challenge in the context of population ageing. It covers different clinical stages and conditions, including mild cognitive impairment, dementia, and Alzheimer’s disease (Perez Palmer et al., 2022). Patients often show impairments in memory, attention, executive function, and language. These changes are often accompanied by behavioral and psychological symptoms. As a result, cognitive impairment can affect daily living ability, social function, and quality of life (Alfeo et al., 2024). With the rapid ageing of the global population, the number of people living with cognitive impairment continues to increase. This condition has become one of the major causes of disability, long-term care dependence, and death (Livingston et al., 2024). By 2050, the number of people with dementia worldwide is expected to exceed 150 million, which will further increase medical, social, and economic burdens (GBD 2019 Dementia Forecasting Collaborators, 2022). Therefore, exploring safe, effective, and sustainable intervention strategies has become an important direction in the prevention and management of cognitive impairment.

Current treatment for cognitive impairment is still mainly based on pharmacological interventions. However, existing treatments have certain limitations in slowing disease progression and improving long-term outcomes. Non-pharmacological interventions have therefore become an important part of comprehensive management for cognitive impairment. Among these approaches, music therapy is an intervention centered on musical experience and therapeutic interaction. It has been widely used in clinical care and rehabilitation practice for patients with cognitive impairment (Rossi et al., 2024). Through music listening, singing, rhythmic activities, and structured music training, music therapy can evoke emotional responses, promote social interaction, and increase patient engagement (Prick et al., 2024; Baroni Caramel et al., 2024). Previous studies have shown that music interventions may help relieve behavioral and psychological symptoms, such as anxiety, depression, and agitation. They may also have positive effects on maintaining cognitive function and improving quality of life (Gardener et al., 2025; Lin et al., 2023). In patients with mild cognitive impairment and dementia, music-based interventions have shown potential value in improving several cognitive domains, including memory, attention, and executive function (Han et al., 2020). These findings provide important support for the application of music therapy in people with cognitive impairment.

As the clinical value of music therapy has received increasing attention, its potential mechanisms have also gained support from neuroscience research. Studies have shown that musical stimulation can activate broad brain networks related to memory, attention, and emotional processing. This provides a neurobiological basis for the effects of music therapy on cognitive and neuropsychiatric symptoms (Toader et al., 2023; Grossi, 2025). In patients with Alzheimer’s disease and mild cognitive impairment, relatively preserved musical memory is considered one of the important foundations for the therapeutic effects of music interventions (Matziorinis and Koelsch, 2022). In addition, music activities that combine auditory, motor, and social elements may enhance cognitive engagement and social interaction (Chatterjee et al., 2021). These findings offer theoretical support for the use of music therapy in populations with cognitive impairment. With the deepening of related research, the application settings of music therapy have gradually expanded from institutional care to community and home-based environments. Its intervention goals have also extended from symptom relief to cognitive health promotion, functional maintenance, and long-term care support (Lin et al., 2022).

The continued expansion of research scope and application settings has led to a rapid increase in related studies. Music therapy for cognitive impairment has gradually developed into an interdisciplinary research field involving neuroscience, geriatrics, rehabilitation medicine, nursing, psychology, and music therapy (Flo et al., 2022; Raglio et al., 2024). The publication of many randomized controlled trials, systematic reviews, and meta-analyses has provided a relatively rich evidence base for the clinical use of music therapy (Bleibel et al., 2023). However, most existing studies have focused on intervention effects. The knowledge structure, research hotspots, and development trends of this field have not been systematically examined. Bibliometrics, through quantitative analysis and knowledge map visualization, can reveal patterns of disciplinary development, research hotspots, and academic collaboration. It therefore provides an important perspective for understanding the evolution of a research field (Ninkov et al., 2022). For this reason, a systematic analysis from a macro-level perspective is needed to clarify the knowledge structure, research hotspots, and future directions of music therapy interventions for cognitive impairment.

Based on this background, the present study analyzed publications on music therapy interventions for cognitive impairment from 2006 to 2026. It aimed to systematically examine the global research landscape, knowledge base, hotspot evolution, and development trends in this field. In addition, randomized controlled trials were analyzed to further evaluate the characteristics of clinical evidence. This study may provide useful references for future research design, clinical practice, and translational application.

## 2 Materials and methods

### 2.1 Data sources and search strategy

Given the differences among databases in literature coverage, indexing systems, and subject scope, this study used a stratified multi-database search strategy. Records from different databases were not directly merged for analysis. The Web of Science Core Collection (WoSCC) was selected as the main data source for the bibliometric analysis. As one of the most widely used citation databases, WoSCC provides a well-established citation indexing system. It is also highly compatible with mainstream bibliometric tools, such as Bibliometrix, CiteSpace, and VOSviewer. Therefore, it is suitable for visual analyses of knowledge structure, research hotspots, and development trends. To reduce the potential bias caused by using a single database and to improve the robustness of the findings, Scopus was also included. Based on the WoSCC analysis, Scopus was used to cross-validate key indicators, including publication growth trends, country or regional distribution, and major contributing institutions.

Research on music therapy interventions for cognitive impairment involves both the evolution of academic knowledge and the accumulation of clinical intervention evidence. For this reason, randomized controlled trials (RCTs) in PubMed were also searched and analyzed. PubMed has a standardized Medical Subject Headings (MeSH) indexing system and strict literature inclusion standards. It is therefore highly useful for identifying clinical evidence (Williamson and Minter, 2019). By combining subject-related search terms with publication type restrictions, representative RCTs in this field can be identified with relatively high accuracy. This provides additional evidence for understanding the clinical application and translational value of major research topics. By integrating results from WoSCC, Scopus, and PubMed, this study assessed the development of the field from two perspectives: knowledge evolution and clinical application.

The WoSCC search was completed on May 31, 2026. The time span was set from January 1, 2006 to May 31, 2026. The search strategy consisted of music therapy-related terms (TS1) and cognitive impairment-related terms (TS2): TS1 = (“music therapy” OR “music intervention*” OR “music-based intervention*” OR “therapeutic music” OR “music listening” OR “music medicine” OR “musical intervention*” OR “singing intervention*” OR “singing therap*”); TS2 = (“cognitive impairment” OR “mild cognitive impairment” OR “cognitive decline” OR “cognitive dysfunction” OR “cognitive disorder*” OR dementia OR “Alzheimer disease” OR “Alzheimer’s disease”). The final search formula was TS = TS1 AND TS2. During literature screening, only English-language Articles and Reviews were included. Conference abstracts, editorial materials, book chapters, news items, corrections, and other non-research publications were excluded.

To examine the consistency of results across databases, Scopus was searched at the same time. A search strategy broadly consistent with that used in WoSCC was applied in the TITLE-ABS-KEY field to ensure comparability (Singh et al., 2021).

PubMed was used to retrieve RCTs and to evaluate the development of clinical research on music therapy interventions for cognitive impairment. The search was conducted up to May 31, 2026. The search formula was as follows: (“music therapy” OR “music intervention” OR “music-based intervention” OR “therapeutic music” OR “music listening” OR “singing intervention”) AND (“cognitive impairment” OR “mild cognitive impairment” OR “cognitive decline” OR “cognitive dysfunction” OR “cognitive disorder” OR dementia OR “Alzheimer disease” OR “Alzheimer’s disease”) AND (“randomized controlled trial”[Publication Type] OR randomized[Title/Abstract]) AND (“2006/01/01”[Date - Publication]: “2026/05/31”[Date - Publication]).

### 2.2 Literature screening process

The literature screening process is shown in Figure 1A. The initial search in WoSCC identified 1,028 records. After screening, 835 publications were finally included in the bibliometric analysis. Full records and cited references of all included publications were exported and saved in plain text format. To assess the consistency of results across databases, Scopus was searched using the same general search strategy. A total of 1,831 records were retrieved, and 1,368 publications were included after screening for cross-validation analysis. PubMed was used for the supplementary analysis of RCTs. The initial search identified 261 records, and 250 RCTs were finally included after screening. The detailed screening process is provided in Supplementary Material 1. Literature screening and data checking were performed independently by two researchers. Any disagreement was resolved through discussion.

**Figure 1:**
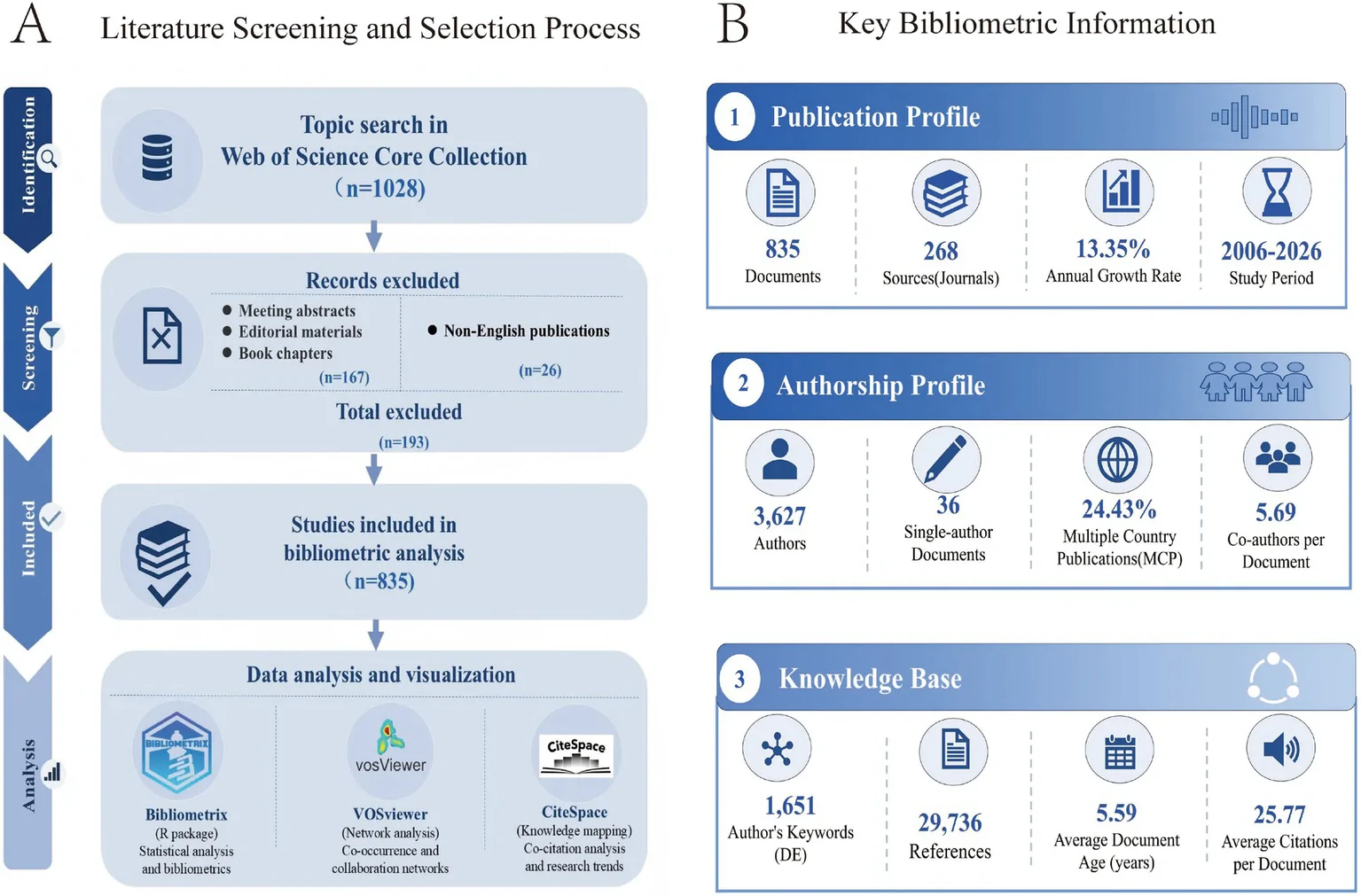
Literature screening and bibliometric overview of music therapy research on cognitive impairment. **(A)** Literature screening process based on the WoSCC database. **(B)** Main bibliometric characteristics of the included publications.

### 2.3 Data analysis

The publications retrieved from WoSCC were used as the main dataset. Bibliometric analysis and visualization were conducted using Bibliometrix (v5.0.1), CiteSpace (v6.4. R1), and VOSviewer (v1.6.20) (Chen, 2018; Caneppele et al., 2023). Bibliometrix was used for basic statistical analysis, collaboration network construction, and thematic structure analysis. CiteSpace was used for citation burst detection, temporal evolution analysis, and identification of the knowledge base. VOSviewer was used to construct and visualize co-occurrence, co-citation, and collaboration networks. In this study, multiple-country publications (MCPs) were defined as publications in which the authors’ affiliations came from two or more countries or regions.

In the Bibliometrix analysis, automatic layout optimization was used to improve the network structure, and the Walktrap algorithm was applied to identify thematic clusters. Keyword co-occurrence analysis was normalized using the Association Strength method. Isolated nodes and weakly related terms were removed, and only high-frequency keywords were retained for analysis. In CiteSpace, the time slice was set to 1 year. The Pathfinder algorithm was used for network pruning, and nodes were selected based on the g-index with k = 25. These settings were used to identify research frontiers and patterns of knowledge evolution. In VOSviewer, the Fractional Counting method was used to construct networks. The minimum occurrence threshold for keywords was set at five.

Data from Scopus were mainly used for cross-validation of key results. RStudio was used to analyze publication trends, country or regional distribution, and institutional contributions. These analyses were conducted to assess the consistency of results across databases. RCTs retrieved from PubMed were analyzed using Bibliometrix for descriptive statistics and thematic analysis. This analysis was used to supplement the understanding of development trends and research topics in clinical intervention studies.

In addition, Microsoft Excel 2024 was used for data organization and statistical processing. Adobe Illustrator 2026 was used for figure refinement and final editing.

## 3 Results

### 3.1 General characteristics and publication trends

A total of 835 publications were finally included in the analysis. These publications were published in 268 academic journals and involved 3,627 authors. Internationally co-authored publications accounted for 24.43% of all records. Each publication had an average of 5.69 authors. The average annual growth rate during the study period was 13.35% (Figure 1B).

The annual number of publications increased from 4 in 2006 to 102 in 2025, showing a clear upward trend. From 2006 to 2016, research output grew slowly. After 2017, the annual number of publications increased markedly and remained at a relatively high level after 2020. The cumulative number of publications also continued to rise. The fitted curve was generally consistent with the observed trend, reflecting the long-term growth pattern of publications in this field (Table 1, Figure 2A). The prediction interval shown in Table 1 gradually widened over time. This indicates greater uncertainty in the model estimates for later publication trends. This interval was used to describe the uncertainty range of model prediction, rather than the statistical confidence interval of the actual annual publication counts.

**Table 1:** Observed publication counts and model-based 95% prediction intervals for annual publication output (2006–2026).

Year	Articles	Lower_PI	Upper_PI
2006	4	2.7042	7.1008
2007	4	3.2606	8.4673
2008	5	3.9291	10.1023
2009	8	4.732	12.0601
2010	11	5.6956	14.4059
2011	10	6.8513	17.2184
2012	13	8.2363	20.5929
2013	16	9.8949	24.6443
2014	25	11.8801	29.5117
2015	22	14.2542	35.3634
2016	25	17.0916	42.4029
2017	43	20.4806	50.8767
2018	62	24.5256	61.0835
2019	39	29.3508	73.385
2020	67	35.103	88.2197
2021	71	41.9563	106.1194
2022	82	50.1171	127.7288
2023	84	59.8296	153.8302
2024	93	71.383	185.3727
2025	102	85.1196	223.5084
2026*	49	101.4446	269.6355

PI, prediction interval.

The observed publication counts from 2006 to 2025 represent complete calendar years. The observed count for 2026 includes 49 publications indexed from January 1 to May 31, 2026 only. The exponential model was fitted using complete-year data from 2006 to 2025. Accordingly, the 95% PI reported for 2026 represents the predicted range of publication counts for the full 2026 calendar year and is distinct from the partial-year observed count of 49. The partial-year observed count and the full-year model-based prediction interval should not be directly compared. *indicates that the literature data for that year are incomplete because the literature search covered only part of the year.

**Figure 2:**
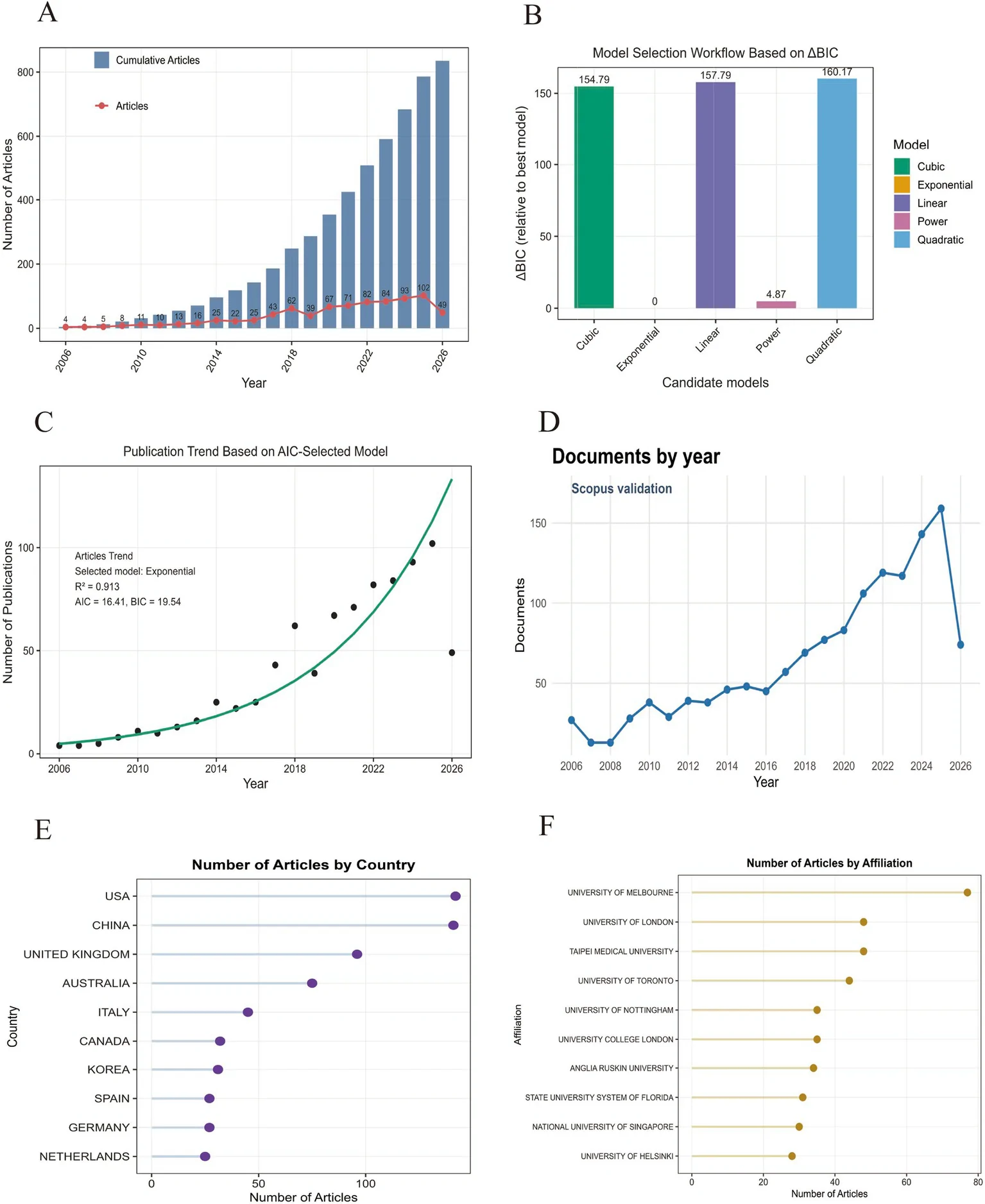
Publication trends and geographic distribution**. (A)** Annual and cumulative publication trends in WoSCC. **(B)** Model selection based on ΔBIC. **(C)** Fitted trend using the AIC-selected exponential model. **(D)** Annual publication trend in Scopus. **(E)** Top 10 contributing countries. **(F)** Top 10 contributing institutions.

The comparison of different functional models showed that both the Bayesian information criterion (BIC) and the Akaike information criterion (AIC) supported the exponential model as the best-fitting model (Figures 2B,C). The exponential model was highly consistent with the observed annual publication trend (*R*^2^ = 0.913). This suggests a clear pattern of cumulative growth in research output in this field. Cross-validation based on the Scopus database further showed that the annual publication trend was generally consistent with the WoSCC results (Figure 2D). Both databases showed a sustained increase in publication output.

### 3.2 Contributions of countries or regions and institutions

The distribution of publications by country or region showed that research on music therapy interventions for cognitive impairment has developed into a broad international field (Table 2, Figure 2E). The United States ranked first with 142 publications, followed closely by China with 141 publications. Together, these two countries contributed nearly one-third of all publications. The United Kingdom ranked third with 96 publications, followed by Australia with 75 publications. Overall, research activity was mainly concentrated in North America, Europe, and the Asia-Pacific region.

**Table 2:** Top 10 countries/regions with the highest productivity and specific parameters.

Country	Articles	Articles %	SCP	MCP	MCP %
USA	142	17.0	125	17	11.9
China	141	16.8	117	24	17.0
United Kingdom	96	11.4	76	20	20.8
Australia	75	8.9	51	24	32
Italy	45	5.3	36	9	20
Canada	32	3.8	26	6	18.7
Korea	31	3.7	26	5	16.1
Germany	27	3.2	21	6	22.2
Spain	27	3.2	23	4	14.8
Netherlands	25	2.9	14	11	44

SCP, Single Country Publications; MCP, Multiple Country Publications.

In terms of multiple-country publications, China and Australia each published 24 internationally collaborative papers. The United Kingdom and the United States published 20 and 17, respectively. When calculated as the proportion of multiple-country publications among total national output, the Netherlands ranked among the highest at 44.0%, followed by Australia at 32.0%. The corresponding proportion for the United States was 11.9%.

The country-level research pattern was also reflected in the distribution of institutions (Table 3, Figure 2F). The University of Melbourne ranked first with 77 publications. Taipei Medical University and the University of London each published 48 papers, followed by the University of Toronto with 44 publications. High-output institutions were mainly located in Australia, the United Kingdom, Canada, and China. The Scopus database showed a similar distribution pattern (Supplementary Figure S1). The University of Melbourne remained the most productive institution. The University of Toronto, the University of London, the University of Nottingham, and Anglia Ruskin University also ranked among the leading institutions.

**Table 3:** Top 10 institutions ranked by publication output.

Affiliation	Articles
University of Melbourne	77
Taipei Medical University	48
University of London	48
University of Toronto	44
University College London	35
University of Nottingham	35
Anglia Ruskin University	34
State University System of Florida	31
National University of Singapore	30
University of Helsinki	28

### 3.3 Collaboration networks and knowledge base

To reveal the research communities and knowledge base of music therapy interventions for cognitive impairment, collaboration networks of authors, countries, and institutions were constructed. Co-citation networks of authors and journals were also analyzed (Figure 3).

**Figure 3:**
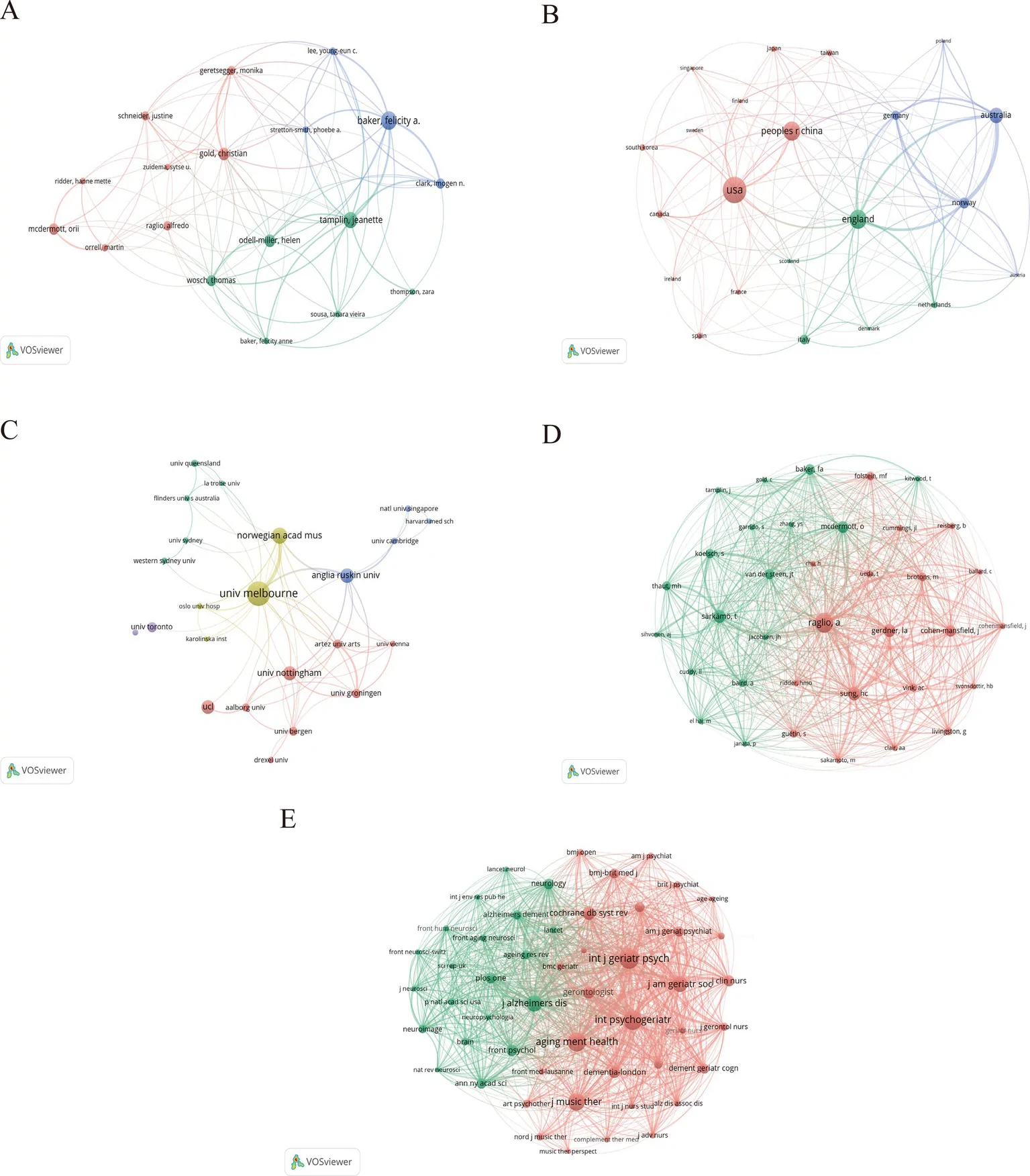
Collaboration networks and intellectual structure of music therapy research on cognitive impairment. **(A)** Author collaboration network. **(B)** Country collaboration network. **(C)** Institutional collaboration network. **(D)** Author co-citation network. **(E)** Journal co-citation network.

The author collaboration network showed that several relatively stable research teams have formed in this field (Figure 3A). Baker Felicity A., Tamplin Jeanette, Gold Christian, and Odell-Miller Helen were located at the core of the network. Collaborations were mainly concentrated within individual research teams, while links between different groups were relatively limited. In the country collaboration network, the United States, China, the United Kingdom, and Australia formed the core of the international collaboration system (Figure 3B). The United States played an important role in connecting research groups in Europe and the Asia-Pacific region. The institutional collaboration network showed that the University of Melbourne occupied a central position and maintained close collaborations with the Norwegian Academy of Music, Anglia Ruskin University, and the University of Nottingham (Figure 3C).

The author co-citation network revealed the main knowledge base of this field (Figure 3D). Raglio A., Baker F. A., Särkämö T., Sung H. C., and Cohen-Mansfield J. had high co-citation frequencies. Among them, Raglio A. and Baker F. A. showed particularly strong influence. Several closely connected knowledge clusters were identified in the co-citation network. These clusters mainly focused on music interventions, neurorehabilitation, cognitive function improvement, and the management of behavioral and psychological symptoms. Journal co-citation analysis showed that International Psychogeriatrics, International Journal of Geriatric Psychiatry, Aging & Mental Health, and Journal of Alzheimer’s Disease were important knowledge sources in this field (Figure 3E). In addition, neuroscience and geriatric medicine journals, such as Neurology, Alzheimer’s & Dementia, and Frontiers in Aging Neuroscience, showed broad links with music therapy-related journals. This pattern reflects the clear interdisciplinary nature of the field.

### 3.4 Research hotspots and thematic evolution

The keyword co-occurrence network showed the core topics and their relationships in research on music therapy interventions for cognitive impairment (Figure 4A). “Dementia,” “music therapy,” “music,” and “Alzheimer’s disease” were located at the center of the network. These keywords were closely connected with “quality of life,” “depression,” “agitation,” and “cognitive impairment.” This indicates that research in this field mainly focuses on dementia, Alzheimer’s disease, cognitive function, behavioral and psychological symptoms, and quality of life. Keywords such as “caregiver,” “nursing home,” “rehabilitation,” and “mental health” were distributed around the core topics. These findings show that long-term care, rehabilitation management, and mental health support are also important research areas.

**Figure 4:**
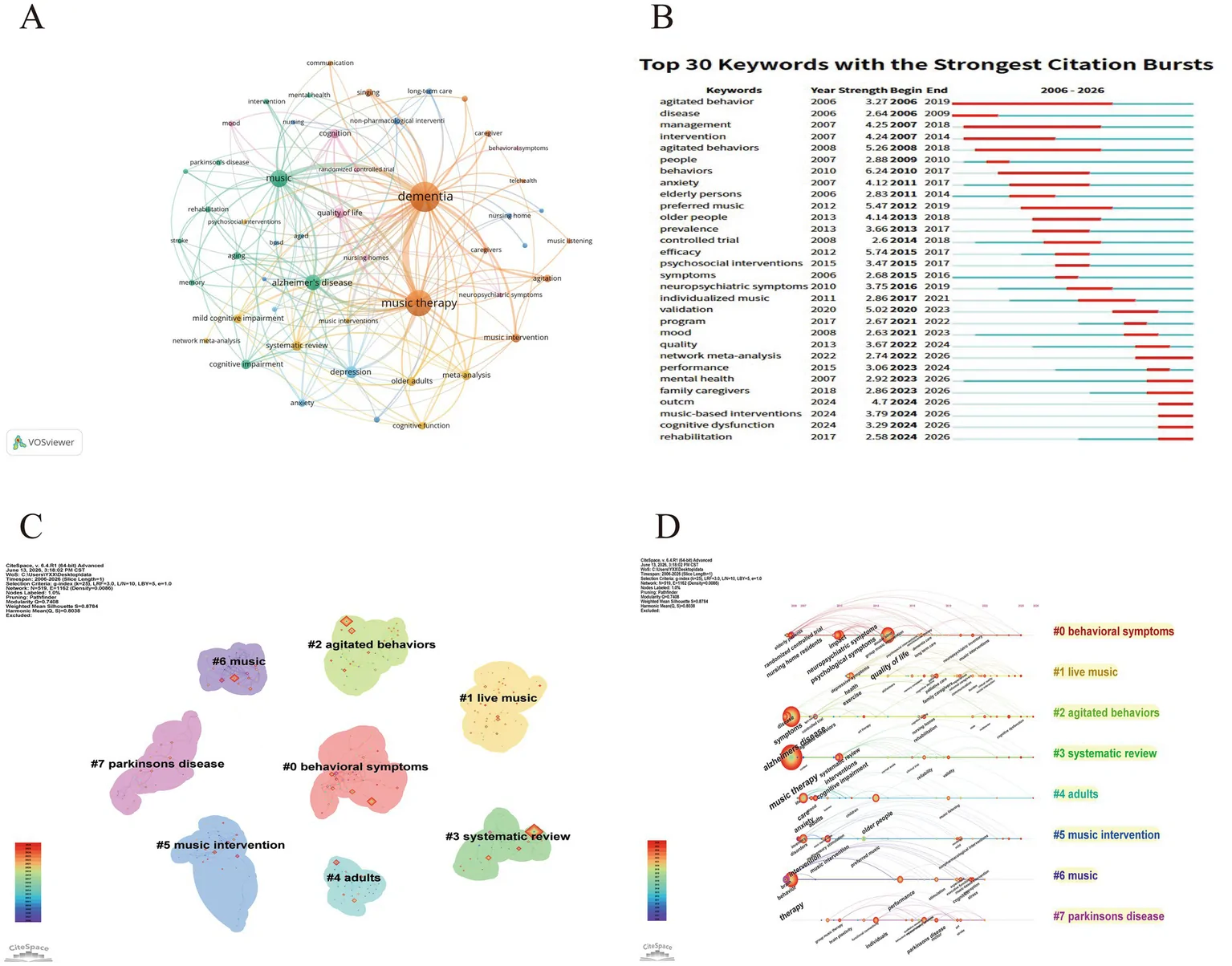
Keyword co-occurrence, burst detection, and thematic evolution. **(A)** Keyword co-occurrence network. **(B)** Top 30 keywords with the strongest citation bursts. **(C)** Keyword clustering analysis. **(D)** Timeline visualization of keyword clusters.

Keyword burst analysis showed stage-specific changes in research hotspots (Figure 4B). Early burst keywords included “agitated behavior” “management” “behaviors” and “anxiety.” These terms indicate that early studies mainly focused on behavioral symptoms and the management of neuropsychiatric symptoms. Later keywords such as “preferred music” “individualized music” “controlled trial” and “efficacy” remained active. This suggests growing attention to individualized music interventions and clinical effect evaluation. In recent years keywords such as “mental health” “family caregivers” “outcomes” “music-based interventions” “cognitive dysfunction” and “rehabilitation” have emerged. The research focus has further expanded to mental health caregiver involvement cognitive outcomes and rehabilitation applications.

Keyword clustering analysis identified the major knowledge modules in this field (Figure 4C). The clusters mainly centered on symptom management in dementia and Alzheimer’s disease forms of music intervention cognitive function assessment long-term care settings and evidence-based research. Together these clusters formed a thematic structure composed of disease populations intervention approaches outcome assessment and application settings. Timeline analysis further showed the continuity and overlap of different themes (Figure 4D). Management of behavioral and psychological symptoms was more prominent in the early stage. Later topics related to music intervention models cognitive function assessment and quality of life continued to develop. In recent years care support cognitive rehabilitation and mental health have become more closely connected with existing research directions.

### 3.5 Thematic structure and development trends

Thematic evolution analysis showed clear thematic continuity in research on music therapy interventions for cognitive impairment (Figure 5A). From 2006 to 2021, “dementia” and “Alzheimer’s disease” formed the main thematic paths. These themes continued into the period from 2022 to 2026. During the same period, early themes related to “depression” and “therapy” gradually became linked with “older adults” and “cognitive impairment.” This suggests that the research focus expanded from dementia and Alzheimer’s disease to older adults and cognitive outcome assessment.

**Figure 5:**
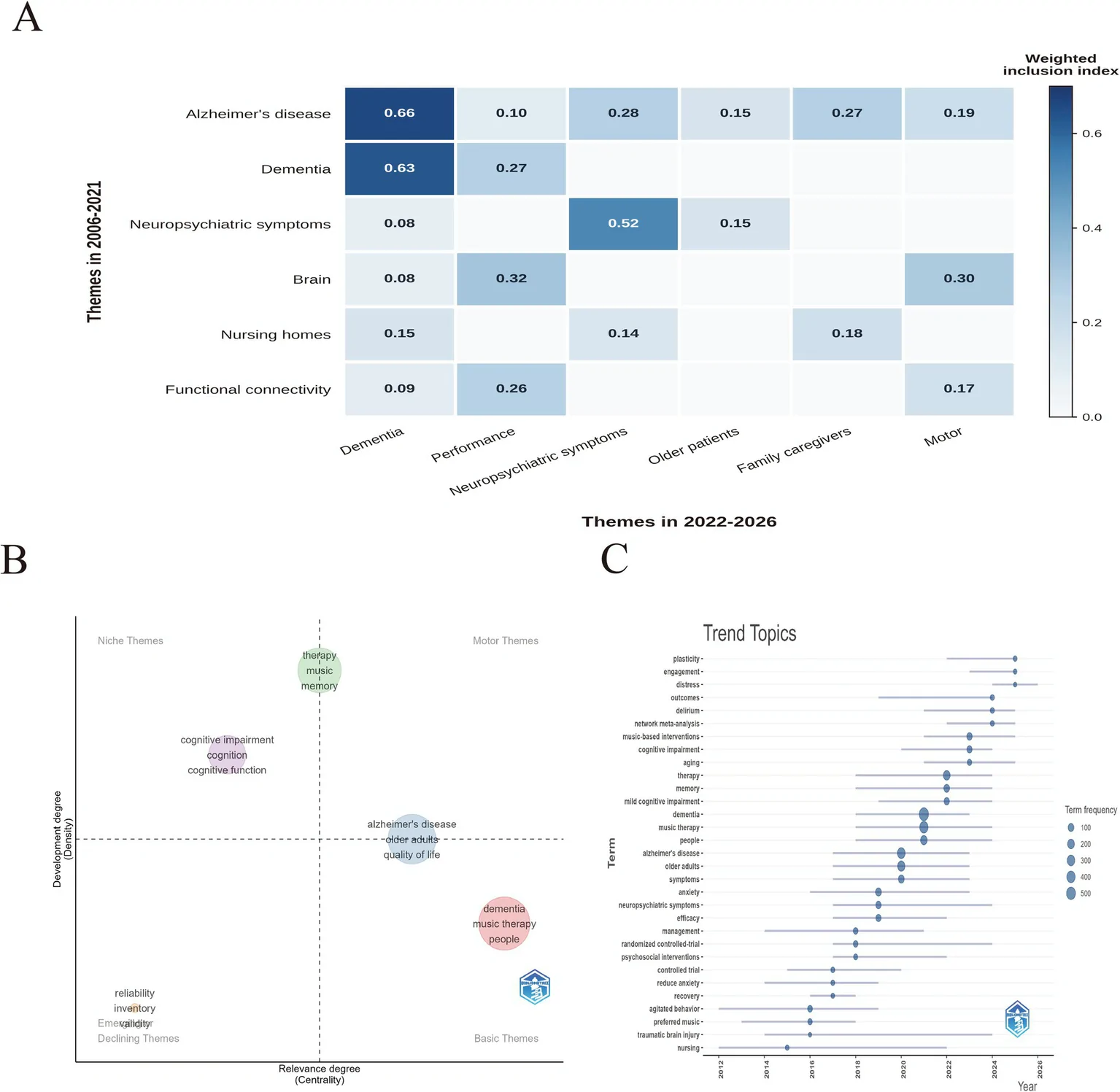
Thematic structure and trend evolution. **(A)** Thematic evolution map. **(B)** Thematic map. **(C)** Trend topics over time.

The thematic map showed the distribution of different themes within the research structure (Figure 5B). “Dementia,” “music therapy,” and “therapy” were located in the basic-theme area and showed high relevance. These terms represented stable core themes in the field. “Alzheimer’s disease,” “music therapy,” and “quality of life” were close to the center of the map, indicating their links with multiple research topics. In contrast, “memory” and “mild cognitive impairment” were located in the niche-theme area. These topics were relatively focused, but their connections with the basic themes were more limited.

Trend topic analysis showed changes in research themes over time (Figure 5C). Early topics included “agitated behavior,” “nursing,” “management,” and “preferred music.” These studies mainly focused on behavioral symptom management, nursing practice, and individualized music use. In the middle stage, topics such as “anxiety,” “depression,” “neuropsychiatric symptoms,” and “quality of life” became more active. Research outcomes expanded from symptom-related indicators to psychological status and quality of life. In recent years, “cognitive impairment,” “family caregivers,” “engagement,” “music-based interventions,” and “plasticity” have continued to appear. These topics indicate growing attention to cognitive function, caregiver involvement, music intervention models, and potential mechanisms.

### 3.6 Citation impact and core publications

The analysis of highly cited publications revealed the academic influence and core literature sources of music therapy interventions for cognitive impairment. The bubble plot of the top 10 globally cited publications showed the relationship between total citation frequency and journal influence (Figure 6A). Several classic studies maintained high citation levels over a long period. Local citation analysis identified representative publications that had important influence on the development of this field (Figure 6B). These studies formed an important basis for knowledge dissemination and academic exchange within the field.

**Figure 6:**
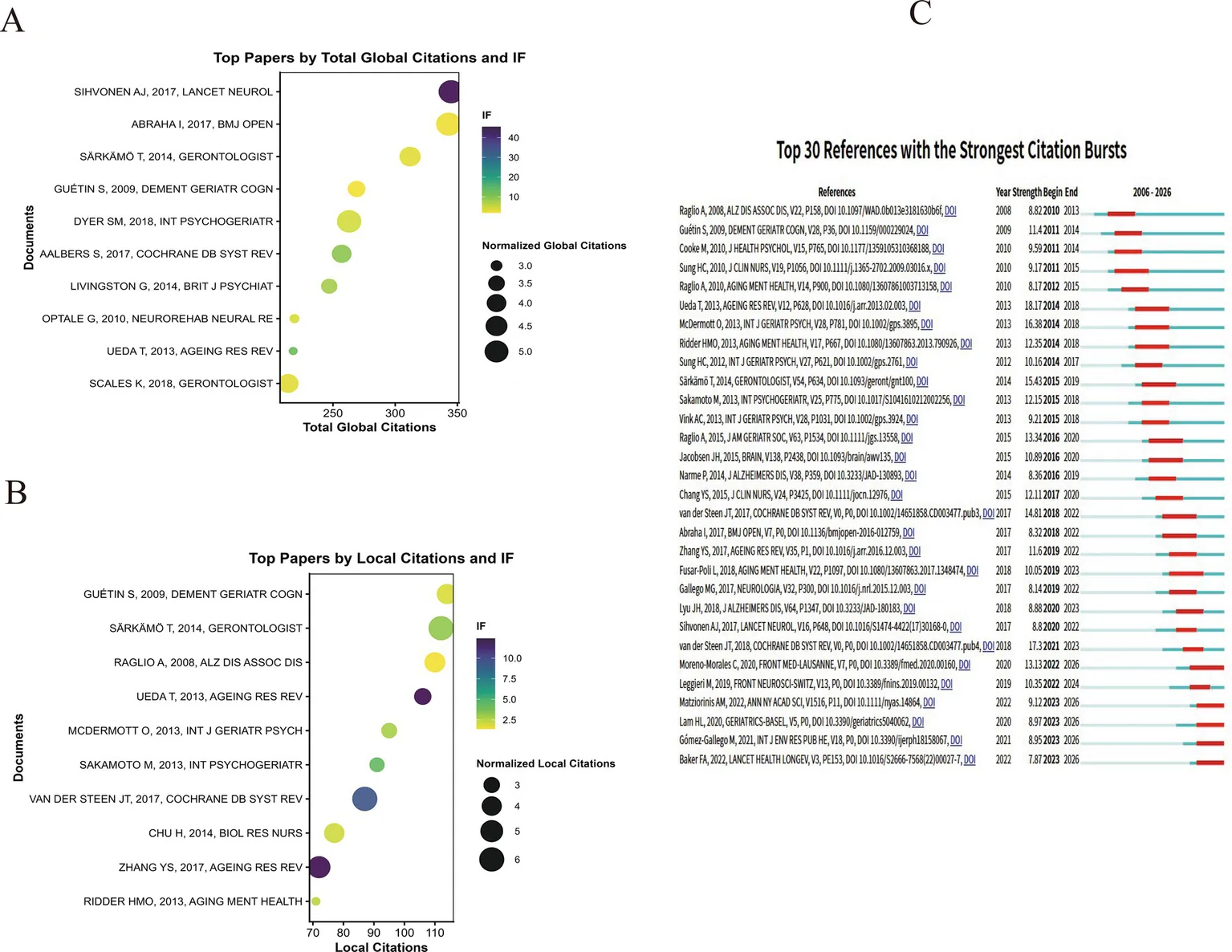
Citation impact and core literature. **(A)** Top 10 globally cited documents. **(B)** Top 10 locally cited documents. **(C)** Top 30 references with the strongest citation bursts.

Reference burst analysis identified 30 publications with strong citation burst intensity (Figure 6C). These publications reflected the changing research focus at different stages. Early burst references mainly focused on cognitive function assessment tools and the use of music interventions in patients with dementia. Examples include studies published by Raglio, Guétin, and Cooke between 2008 and 2010. These studies showed long burst durations and laid an important foundation for applying music therapy to cognitive impairment. After 2017, research attention gradually shifted toward evidence construction and intervention effect evaluation. Systematic reviews and meta-analyses began to receive increasing attention. In recent years, studies by Moreno-Morales et al. (2020), Matziorinis and Koelsch (2022), Lam et al. (2020), and Baker et al. (2022) have shown citation bursts that continued until 2026. This suggests that music therapy remains a current frontier in cognitive function maintenance, healthy ageing, and clinical translation.

The analysis of author academic impact showed that several core research teams have made sustained contributions to this field (Table 4). TAMPLIN Jeanette had the highest *h*-index (*h* = 12) and also showed a high *g*-index (*g* = 21), indicating strong and sustained academic influence. BAKER Felicity A. and GOLD Christian had particularly high total citation counts, with 464 and 676 citations, respectively. In addition, MCDERMOTT Orii and RAGLIO Alfredo had relatively fewer publications but maintained high academic influence. This suggests that research quality and academic contribution in this field do not depend solely on publication volume.

**Table 4:** Ranking of academic quantitative indicators of the top 10 authors.

Author	h-index	g-index	m-index	TC	NP	PY_start
Tamplin Jeanette	12	21	1.33	447	23	2018
Baker Felicity A.	11	21	0.55	464	27	2007
Gold Christian	9	17	0.64	676	17	2013
Mcdermott Orii	9	15	0.64	506	15	2013
Clark Imogen N.	8	13	0.88	227	13	2018
Raglio Alfredo	8	12	0.42	525	12	2008
Lee Young-Eun C.	7	9	0.77	224	9	2018
Odell-Miller Helen	7	16	0.58	269	17	2015
Orrell Martin	7	9	0.5	454	9	2013
Ridder Hanne Mette	7	8	0.5	461	8	2013

TC, Total Citations; NP, Number of Papers; PY, Publication Year.

The ranking of highly cited publications further revealed the core knowledge base of the field (Table 5). The most cited publications included the study by Guétin et al. in Dementia and Geriatric Cognitive Disorders with 114 citations, the study by Särkämö et al. in The Gerontologist with 112 citations, and the study by Raglio et al. in Alzheimer Disease and Associated Disorders with 110 citations. These studies mainly focused on the effects of music therapy on cognitive function, behavioral symptoms, and quality of life in patients with dementia. In addition, several systematic reviews, randomized controlled trials, and evidence-based medicine studies also ranked among the highly cited publications.

**Table 5:** Ranking of the top 20 cited papers and detailed information.

Cited references	Total citations
Guétin S, 2009, Dement Geriatr Cogn, V28, P36, DOI: 10.1159/000229024	114
Särkämö T, 2014, Gerontologist, V54, P634, DOI: 10.1093/GERONT/GNT100	112
Raglio A, 2008, ALZ DIS ASSOC DIS, V22, P158, DOI: 10.1097/WAD.0B013E3181630B6F	110
Folstein MF, 1975, J Psychiat Res, V12, P189, DOI: 10.1016/0022-3956(75)90026-6	109
Ueda T, 2013, Ageing Res Rev, V12, P628, DOI: 10.1016/J.ARR.2013.02.003	106
Mcdermott O, 2013, Int J Geriatr Psych, V28, P781, DOI: 10.1002/GPS.3895	95
Jacobsen JH, 2015, Brain, V138, P2438, DOI: 10.1093/BRAIN/AWV135	91
Sakamoto M, 2013, Int Psychogeriatr, V25, P775, DOI: 10.1017/S1041610212002256	91
Van Der Steen JT, 2017, COCHRANE DB SYST REV, DOI: 10.1002/14651858. CD003477. PUB3	87
Chu H, 2014, Biol Res Nurs, V16, P209, DOI: 10.1177/1099800413485410	77
Gerdner LA, 2000, Int Psychogeriatr, V12, P49, DOI: 10.1017/S1041610200006190	75
Zhang YS, 2017, Ageing RES Rev, V35, P1, DOI: 10.1016/J.ARR.2016.12.003	72
Raglio A, 2015, J Am Geriatr Soc, V63, P1534, DOI: 10.1111/JGS.13558	71
Ridder HMO, 2013, Aging Ment Health, V17, P667, DOI: 10.1080/13607863.2013.790926	71
Van Der Steen JT, 2018, Cochrane DB Syst Rev, DOI: 10.1002/14651858.CD003477. PUB4	71
Svonsdottir HB, 2006, Int Psychogeriatr, V18, P613, DOI: 10.1017/S1041610206003206	70
Sung HC, 2010, J Clin Nurs, V19, P1056, DOI: 10.1111/J.1365-2702.2009.03016	67
Cummings JL, 1994, Neurology, V44, P2308, DOI: 10.1212/WNL.44.12.2308	60
Sung HC, 2012, Int J Geriatr Psych, V27, P621, DOI: 10.1002/GPS.2761	59
Irish M, 2006, Dement Geriatr Cogn, V22, P108, DOI: 10.1159/000093487	58

### 3.7 Clinical evidence landscape and development directions of randomized controlled trials

A total of 250 RCTs were included from the PubMed database. These studies were published in 130 journals and involved 1,225 authors. The average annual growth rate was 6.46%, indicating the continued development of clinical research on music therapy interventions for cognitive impairment. Internationally co-authored publications accounted for 22.8%, suggesting an active transnational collaboration network in this field (Figure 7A).

**Figure 7:**
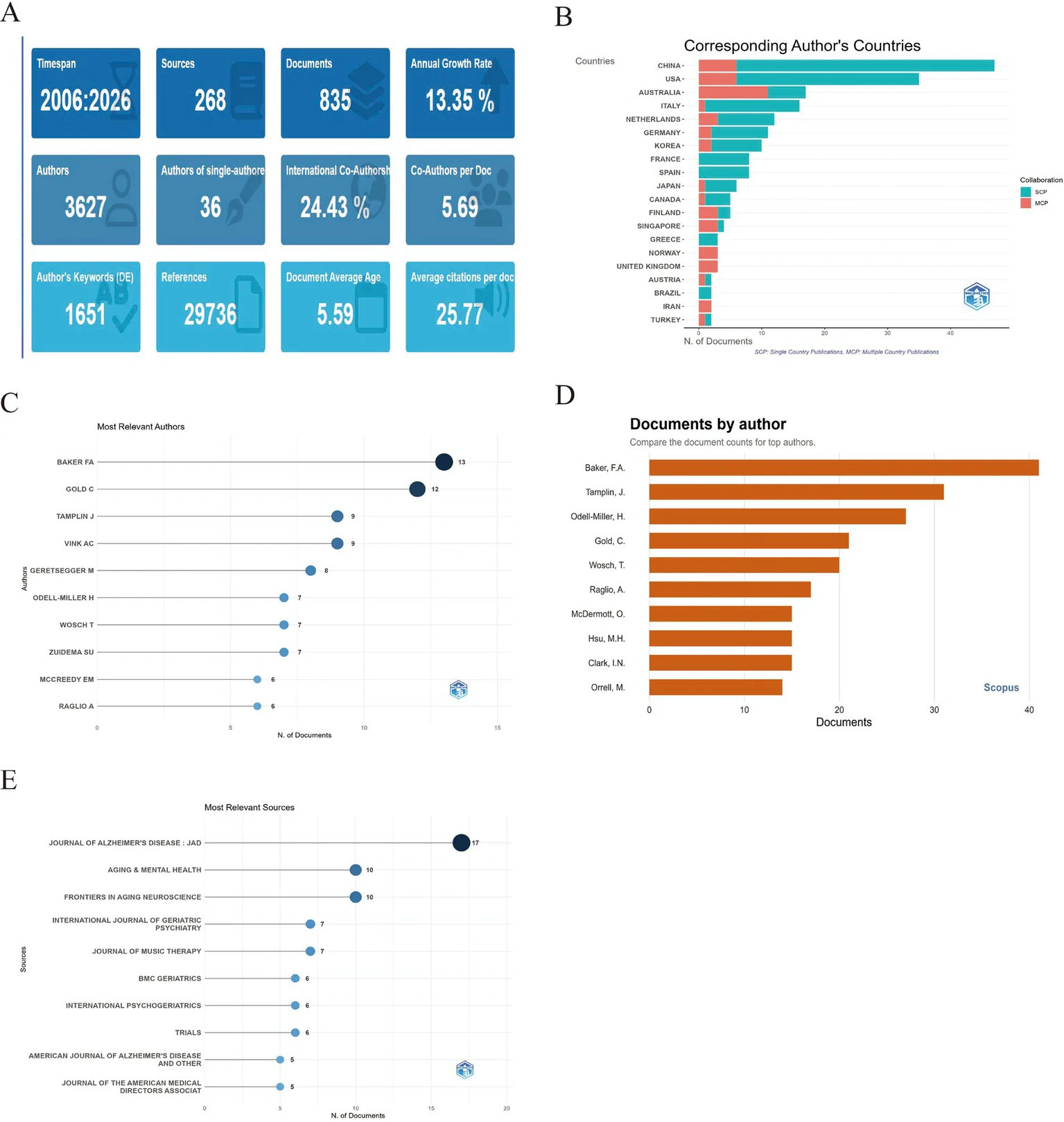
Overview of randomized controlled trials on music therapy for cognitive impairment. **(A)** Main bibliometric information of RCTs from PubMed. **(B)** Country distribution. **(C)** Most productive authors. **(D)** Comparison of active authors between PubMed RCTs and Scopus records. **(E)** Most productive journals.

The country distribution showed that China and the United States were the main contributors to RCT research, with publication numbers much higher than those of other countries (Figure 7B). Author analysis showed that Baker FA, Gold C, and Tamplin J were the most active clinical researchers in this field (Figure 7C). Baker FA published the largest number of RCTs in PubMed and also remained highly active among productive authors in Scopus. This indicates her sustained influence in research on music therapy interventions for cognitive impairment (Figure 7D). The journal distribution was mainly concentrated in the fields of geriatric medicine, neuroscience, and mental health. Journal of Alzheimer’s Disease published the largest number of studies, followed by Aging & Mental Health and Frontiers in Aging Neuroscience (Figure 7E).

The keyword co-occurrence network showed the thematic structure of RCTs on music therapy interventions for cognitive impairment (Figure 8A). Keywords in the network were mainly distributed around study populations, music therapy interventions, clinical symptom assessment, quality of life, and treatment outcomes. Many links were observed among different themes. This indicates a certain degree of connection among RCT research topics. The studies covered patient characteristics, intervention approaches, and multidimensional outcome assessment.

**Figure 8:**
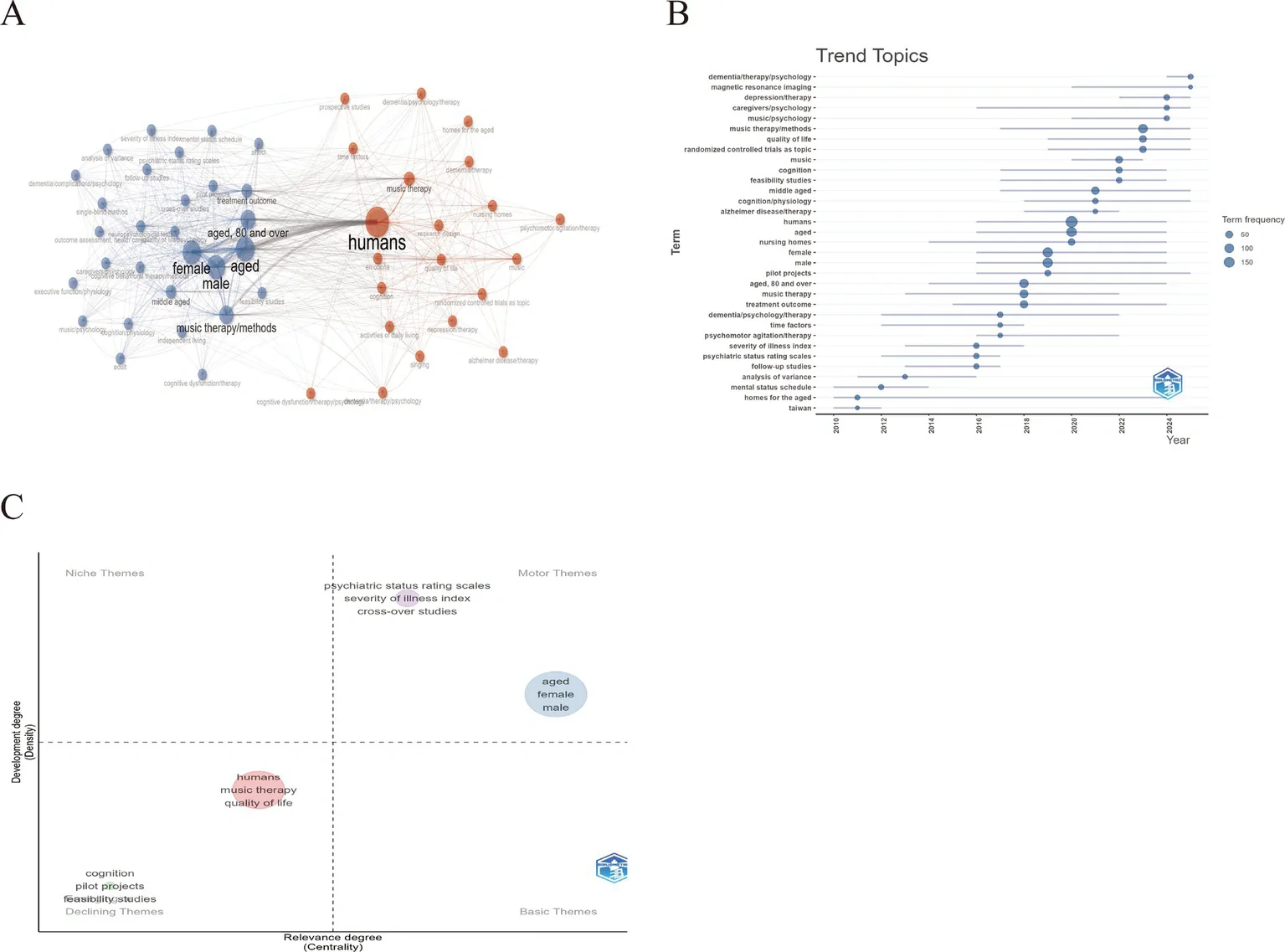
Keyword structure and thematic trends of randomized controlled trials. **(A)** Keyword co-occurrence network of RCTs. **(B)** Trend topics over time. **(C)** Thematic map of RCT-related keywords.

Trend topic analysis showed stage-specific changes in RCT research hotspots over time (Figure 8B). Early themes mainly included “mental status schedule,” “homes for the aged,” “analysis of variance,” “psychiatric status rating scales,” “severity of illness index,” and “psychomotor agitation/therapy.” These topics were mainly related to clinical assessment, care settings, and symptom-related outcomes. Around 2018, topics such as “music therapy,” “aged, 80 and over,” “treatment outcome,” “male,” “female,” “nursing homes,” and “Alzheimer disease/therapy” became more active. Study populations and disease-related interventions gradually became important research contents. In recent years, “quality of life,” “music therapy/methods,” “caregivers/psychology,” “depression/therapy,” and “dementia/therapy/psychology” have continued to appear. The research scope has further extended to quality of life, caregiver psychology, and mental health outcomes. At the same time, the emergence of “magnetic resonance imaging” suggests that some RCTs have begun to include imaging indicators. Mechanism-related evaluation has gradually entered the research field.

The thematic map showed the distribution of RCT research topics (Figure 8C). “Aged,” “female,” and “male” were located in the motor-theme quadrant, with high centrality and maturity. “Psychiatric status rating scales,” “severity of illness index,” and “cross-over studies” were also located in high-density areas. This indicates that topics related to clinical symptom assessment and trial design were relatively mature. “Humans,” “music therapy,” and “quality of life” were located in the low-centrality and low-density area. “Cognition,” “pilot projects,” and “feasibility studies” were also concentrated in this area. These findings suggest that topics related to cognitive outcomes, feasibility studies, and quality of life remain relatively scattered in RCT research.

## 4 Discussion

### 4.1 Overall research landscape

Using a stratified multi-database strategy based on WoSCC, Scopus, and PubMed, this study systematically examined the development of music therapy interventions for cognitive impairment from two perspectives: knowledge structure and clinical evidence. The results showed that research in this field has continued to grow over the past two decades and entered a faster stage of development after 2017. The exponential model also suggested a clear pattern of knowledge accumulation. Publication trends, collaboration networks, and the development of RCTs showed good consistency. These findings suggest that music therapy for cognitive impairment has gradually moved beyond early exploration and has formed a relatively stable research base and academic community.

The country and institution analyses showed that the United States, China, the United Kingdom, and Australia are the main contributors to this field. China and the United States made important contributions to both total publication output and RCT production, suggesting that this topic has received broad attention across different regions. Australia showed strong performance in institutional influence, author contribution, and international collaboration. The University of Melbourne has long occupied a central position in the institutional collaboration network. High-impact scholars such as Baker and Tamplin are also mainly from Australian research teams. This reflects a relatively stable research system in Australia at the intersection of music therapy and cognitive impairment. The United States played an important bridging role in the international collaboration network. Its broad international links may help promote knowledge exchange and resource integration among different research teams.

The author co-citation network, journal co-citation network, and highly cited publication analysis together revealed a relatively stable knowledge base in this field. Scholars such as Raglio, Baker, and Särkämö were important knowledge sources. Journals such as International Psychogeriatrics, International Journal of Geriatric Psychiatry, and Journal of Alzheimer’s Disease provided major academic platforms for the dissemination of research findings. Highly cited publications were mainly randomized controlled trials, systematic reviews, and meta-analyses. This suggests that the evidence base in this field has gradually developed from single clinical observations to evidence-based synthesis. It also provides an important reference for future study design and intervention evaluation.

The 250 randomized controlled trials included from PubMed showed that music therapy interventions for cognitive impairment already have a certain clinical evidence base. RCTs in this field have continued to increase. Their focus has gradually expanded from symptom assessment and treatment effect observation to cognitive function, quality of life, mental health, and care-related outcomes. This change suggests that the clinical value of music therapy is no longer limited to symptom relief. It is increasingly linked to patients’ overall functional status and long-term health benefits. This also provides support for future evidence assessment, intervention optimization, and clinical translation.

### 4.2 Research hotspots and frontier directions

#### 4.2.1 Cognitive improvement and rehabilitation value

This study found that cognitive improvement has remained a core topic in research on music therapy interventions for cognitive impairment. It held an important position in the keyword co-occurrence network, thematic evolution analysis, and RCT evidence map. Dementia and Alzheimer’s disease have long been central themes in this field. This reflects a sustained focus on the key clinical need to slow cognitive decline and maintain functional independence. Cognitive decline is not only a major feature of disease progression. It is also an important cause of functional impairment, increased care dependence, and reduced quality of life (Xing et al., 2024). Given the limited effects of disease-modifying treatments, delaying cognitive decline and preserving existing abilities remain important goals in the management of cognitive impairment (Reetz et al., 2026).

Music activities involve several cognitive processes, including attention, memory, language, executive function, and emotional processing. They can also engage broad brain networks at the same time (Speranza et al., 2022). Meta-analyses have shown that music therapy can improve overall cognitive function in patients with dementia (Ting et al., 2024). Randomized controlled studies have also found that regular singing and music listening may help maintain memory and orientation, and may slow the decline of global cognitive function (Zhou et al., 2025). In addition, the ability of familiar music to evoke autobiographical memory is relatively preserved in patients with Alzheimer’s disease. This may provide a potential neurocognitive basis for the effects of music interventions (Madera-Cimadevilla et al., 2024).

This trend was also reflected in the thematic evolution analysis of the present study. The continued activity of “rehabilitation” suggests that research attention has extended from cognitive improvement to cognitive rehabilitation. Unlike studies that focus only on changes in cognitive scores, cognitive rehabilitation emphasizes the use of preserved abilities. It also focuses on maintaining daily function and social participation. In recent years, several studies have shown that singing training, active music participation, and individualized music listening may improve cognitive performance. They may also support functional maintenance and social engagement (Stekić, 2024; MacRitchie et al., 2020; Saarikallio et al., 2020). Therefore, the value of music therapy in cognitive impairment is expanding from symptom management to rehabilitation support.

#### 4.2.2 Quality of life improvement and care support

Beyond cognitive function, quality of life has gradually become an important outcome for evaluating music therapy interventions. In this study, “quality of life” was closely linked with core keywords such as “dementia,” “Alzheimer’s disease,” and “music therapy.” In recent years, themes such as “caregiver,” “family caregivers,” “engagement,” and “mental health” have remained active. This suggests that research attention has extended from individual patients to long-term care and social support. This change is closely related to the clinical features of cognitive impairment. Patients with cognitive impairment often experience behavioral and psychological symptoms, such as anxiety, depression, agitation, and social withdrawal. These symptoms can weaken social participation and subjective well-being, and they are important causes of reduced quality of life (McCollum and Karlawish, 2020; Hill et al., 2017). As the disease progresses, care needs continue to increase. Patient quality of life and caregiver burden also become closely linked over time (Carlozzi et al., 2018).

Clinical studies have shown that music interventions can improve quality of life in patients with dementia and may have positive effects on emotional problems such as anxiety and depression (Pongan et al., 2017; Zhang et al., 2023; Mei et al., 2025). Community singing and group music activities can promote social participation and emotional communication, thereby improving patients’ lived experience (Lee et al., 2022b). At the same time, the continued activity of themes such as “family caregivers” and “engagement” suggests that music therapy research is no longer limited to patients alone. It is beginning to consider the possible shared benefits for both patients and caregivers. Related studies have shown that music interventions may strengthen emotional connection and interaction quality between patients and caregivers, and may also reduce care-related stress to some extent (Lee et al., 2022a). These findings indicate that the value of music therapy is extending from individual symptom improvement to long-term care support. Its combined effects on cognition, emotion, and social function also provide an important entry point for future mechanistic research.

#### 4.2.3 From efficacy evaluation to neuroplasticity mechanisms

Clinical studies have suggested that music therapy may have positive effects on cognitive function, emotional status, and quality of life. However, its underlying mechanisms remain to be clarified. In the present study, “plasticity” appeared as an emerging theme, and “magnetic resonance imaging” was also observed in the RCT trend topics. These findings suggest that research in this field is extending from clinical effect evaluation to the exploration of neuroplasticity and imaging-based mechanisms.

The introduction of neuroimaging methods provides new tools for observing changes in brain function after music therapy and for exploring possible pathways of action. In recent years, studies using functional magnetic resonance imaging, electroencephalography, and functional near-infrared spectroscopy have shown that music interventions may affect brain network connectivity related to cognitive and emotional processing. They may also promote functional reorganization of the brain (Lyu et al., 2025; Fan et al., 2026; Ding et al., 2024). These findings provide a neurobiological basis for understanding the clinical effects of music therapy. They also move the field from efficacy evaluation toward mechanism explanation.

For patients with Alzheimer’s disease, some neural networks related to music processing and musical memory remain relatively preserved during disease progression (Groussard et al., 2019). Even when memory function is clearly impaired, patients may still recognize familiar music and show related emotional responses (Zhou et al., 2019). Based on this feature, music therapy may work by activating preserved neural networks, promoting functional compensation, and enhancing the adaptive capacity of the nervous system (Zeng et al., 2026). Increasing research on preserved musical memory, changes in brain functional connectivity, and neuroplasticity regulation has provided new evidence for explaining the possible mechanisms of music therapy (Groussard et al., 2019; Rajakumar and Mohan, 2024; Slade et al., 2025).

Nevertheless, current evidence on the mechanisms of music therapy still comes mainly from small-sample neuroimaging studies and exploratory research. Studies differ in intervention forms, observation indicators, and assessment methods. These differences limit the comparison and integration of existing evidence. Future studies may combine functional magnetic resonance imaging, electroencephalography, functional near-infrared spectroscopy, neurotrophic factors, inflammatory markers, and other objective indicators. Such work could help build an evidence chain from brain network changes and biological effects to clinical benefits. It may also provide a theoretical basis for the more precise application of music therapy.

### 4.3 Clinical evidence and translational challenges

This study found that cognitive improvement, quality of life enhancement, and care support have become important research themes in music therapy interventions for cognitive impairment. These themes have also gradually entered the core outcome system of clinical research. The RCT evidence map showed that early studies mainly focused on behavioral and psychological symptoms such as agitation, anxiety, and depression. Later studies gradually expanded to cognitive function, social participation, and overall health outcomes (Guetin et al., 2009; Gallego and García, 2017; Reschke-Hernández et al., 2023). The development of research hotspots was generally consistent with the direction of clinical evidence. This suggests that music therapy research is forming a continuous pathway from the identification of clinical needs to intervention evaluation and application expansion.

Even so, the growth rate of RCTs remains lower than that of the overall literature output. Compared with pharmacological interventions, music therapy is more individualized and context-dependent. Intervention content often needs to be adjusted according to patients’ cognitive status, music preferences, participation ability, and care environment. This increases the difficulty of study design and standardized implementation (Dahms et al., 2021). In addition, studies differ greatly in intervention form, frequency, duration, and outcome measures. Some studies also have small sample sizes and limited long-term follow-up. These factors reduce the comparability of findings and limit evidence synthesis.

With the accumulation of clinical studies, the field is no longer focused only on whether music therapy is effective overall. More attention is now being given to optimizing intervention forms, identifying suitable populations, and improving implementation pathways (Hackett et al., 2022). This change suggests that research on music therapy for cognitive impairment is moving from efficacy verification toward intervention optimization and precise application. Future studies need clearer consensus on intervention standardization, core outcome measures, and long-term effect evaluation. More multicenter randomized controlled trials and real-world studies are also needed to improve the quality of evidence and promote translation into clinical practice.

### 4.4 Strengths and limitations

Compared with previous studies that mainly relied on a single database or bibliometric analysis alone, this study combined bibliometric analysis with evidence from randomized controlled trials. It systematically evaluated music therapy interventions for cognitive impairment from both disciplinary development and clinical application perspectives. The findings describe the evolution of the knowledge structure, research hotspots, and development trends in this field. They also provide a reference for understanding the clinical evidence base and translational pathway of related research.

This study also has several limitations. First, databases differ in literature coverage, citation indexing systems, and metadata standards. Although this study used a unified search strategy and standardized analysis process, differences in database coverage may still have affected the statistics for some countries, institutions, and authors. Second, only English-language publications were included. This may have led to underrepresentation of studies from some non-English-speaking countries and regions. Because the implementation of music therapy is closely related to cultural background, care models, and healthcare systems, language restriction may have affected the complete presentation of the global research landscape to some extent.

In addition, bibliometric analysis mainly uses publication and citation data to construct knowledge maps. It can reflect the general trends and knowledge structure of a field, but it cannot directly assess study design quality, risk of bias, or strength of evidence. Although the RCT analysis added a clinical evidence perspective, several issues still require further systematic evaluation. These include differences in efficacy among music intervention models, suitable populations, core outcomes, and long-term effects.

## 5 Conclusion

Based on the WoSCC, Scopus, and PubMed databases, this study conducted a systematic bibliometric analysis and clinical evidence assessment of music therapy interventions for cognitive impairment from 2006 to 2026. The results showed that publication output in this field has continued to increase, with a faster stage of development emerging in the past decade. The United States, China, the United Kingdom, and Australia were the main contributing countries. Institutions such as the University of Melbourne played an important role in international collaboration and knowledge production. Author and journal co-citation analyses showed that this field has formed a relatively stable knowledge base. The findings also reflected clear interdisciplinary links among neuroscience, geriatric medicine, psychology, and rehabilitation medicine.

The evolution of research hotspots showed that music therapy interventions for cognitive impairment have gradually expanded from early management of behavioral and psychological symptoms to cognitive rehabilitation, mental health promotion, and care support. In recent years, topics such as cognitive function, quality of life, family caregivers, and optimization of music interventions have remained active. This suggests that research goals are moving from symptom relief toward the improvement of overall health outcomes. At the same time, the emergence of new topics such as neuroplasticity indicates a growing interest in explaining the potential mechanisms of music therapy.

The analysis of randomized controlled trials showed that music therapy interventions for cognitive impairment have developed a certain clinical evidence base. Quality of life, cognitive function, and dementia-related psychological problems are important outcomes in current RCT research. However, cognitive outcome assessment, intervention optimization, and feasibility studies still need further attention in future clinical research. More multicenter studies with large samples and long-term follow-up are needed. Future research should also promote the standardization of intervention protocols and core outcome measures. In addition, neuroimaging and biomarker studies should be integrated to clarify underlying mechanisms, thereby supporting the clinical translation and application of music therapy in the management of cognitive impairment.

## Data Availability

Publicly available datasets were analyzed in this study. This data can be found here: the data analyzed in this study were obtained from three bibliographic databases: Web of Science Core Collection, Scopus, and PubMed. The Web of Science Core Collection data were retrieved from https://www.webofscience.com/wos/woscc/basic-search, Scopus data from https://www.scopus.com/search/form.uri, and PubMed data from https://pubmed.ncbi.nlm.nih.gov/. The detailed search strategies, screening process, and inclusion criteria are described in the Methods section and Supplementary material. No repository accession numbers were generated for this study.
