# Supplementary material for: Global trends, research hotspots, and clinical evidence mapping of music therapy interventions for cognitive impairment: a bibliometric and visualization analysis from 2006 to 2026
Source: Front Psychol. 2026 Sep 24;17:1917320. doi: 10.3389/fpsyg.2026.1917320 (PMC13645771; doi:10.3389/fpsyg.2026.1917320)
Supplement: Supplementary file 1 [file Supplementary_file_1.docx]

**Figure S1. Distribution of the top 10 contributing institutions based on publication output in the Scopus database.**

**Supplementary Material 1. Database-specific search strategies, eligibility criteria, screening procedures, reasons for exclusion, and data-processing workflow**

**S1. Overview of the database-specific searches**

**Table S1. Overview of the database-specific searches**

| **Database/index** | **Analytical purpose** | **Search field(s)** | **Search date** | **Publication period** | **Records retrieved, n** | **Records included, n** |
| --- | --- | --- | --- | --- | --- | --- |
| Web of Science Core Collection: Science Citation Index Expanded (SCI-EXPANDED) | Primary bibliometric and visualization analysis | Topic field (TS) | May 31, 2026 | January 1, 2006–May 31, 2026 | 1,028 | 835 |
| Scopus | Independent cross-validation of publication trends and country/region and institutional distributions | Title, Abstract, and Keywords (TITLE-ABS-KEY) | May 31, 2026 | January 1, 2006–May 31, 2026 | 1,831 | 1,368 |
| PubMed | Publication-level descriptive and thematic mapping of randomized controlled trial-related research | Title/Abstract, Publication Type, and Date–Publication | May 31, 2026 | January 1, 2006–May 31, 2026 | 261 | 250 |

**Abbreviations:** WoSCC, Web of Science Core Collection; SCI-EXPANDED, Science Citation Index Expanded; RCT, randomized controlled trial.

**Note:** The 2026 records covered publications indexed from January 1 to May 31, 2026 only. WoSCC, Scopus, and PubMed were analyzed as separate datasets, and no combined publication total was calculated across databases.

**S2. Complete database-specific search strategies**

**Table S2. Complete database-specific search strategies**

| **Database/index** | **Search field(s)** | **Search date and publication period** | **Full search strategy** | **Screening outcome** |
| --- | --- | --- | --- | --- |
| Web of Science Core Collection (WoSCC) Index: Science Citation Index Expanded (SCI-EXPANDED) | Topic field (TS) | Search date: May 31, 2026 Publication period: January 1, 2006-May 31, 2026 | TS=("music therapy" OR "music intervention*" OR "music-based intervention*" OR "therapeutic music" OR "music listening" OR "music medicine" OR "musical intervention*" OR "singing intervention*" OR "singing therap*") AND TS=("cognitive impairment" OR "mild cognitive impairment" OR "cognitive decline" OR "cognitive dysfunction" OR "cognitive disorder*" OR dementia OR "Alzheimer disease" OR "Alzheimer's disease") | Retrieved: 1,028 Excluded: 167 non-eligible publication types; 26 non-English publications Included: 835 |
| Scopus | Title, Abstract, and Keywords (TITLE-ABS-KEY) | Search date: May 31, 2026 Publication period: January 1, 2006-May 31, 2026 | TITLE-ABS-KEY( ("music therapy" OR "music intervention*" OR "music-based intervention*" OR "therapeutic music" OR "music listening" OR "music medicine" OR "musical intervention*" OR "singing intervention*" OR "singing therap*") AND ("cognitive impairment" OR "mild cognitive impairment" OR "cognitive decline" OR "cognitive dysfunction" OR "cognitive disorder*" OR dementia OR "Alzheimer disease" OR "Alzheimer's disease")) AND PUBYEAR > 2005 AND PUBYEAR < 2027 | Retrieved: 1,831 Excluded: 337 non-eligible publication types; 126 non-English publications Included: 1,368 |
| PubMed | Publication Type; Title/Abstract; Date - Publication | Search date: May 31, 2026 Publication period: January 1, 2006-May 31, 2026 | ("music therapy" OR "music intervention" OR "music-based intervention" OR "therapeutic music" OR "music listening" OR "singing intervention") AND ("cognitive impairment" OR "mild cognitive impairment" OR "cognitive decline" OR "cognitive dysfunction" OR "cognitive disorder" OR dementia OR "Alzheimer disease" OR "Alzheimer's disease") AND ("randomized controlled trial"[Publication Type] OR randomized[Title/Abstract]) AND ("2006/01/01"[Date - Publication] : "2026/05/31"[Date - Publication]) | Retrieved: 261 Excluded after eligibility assessment: 11 Included: 250 |

**Abbreviations:** WoSCC, Web of Science Core Collection; SCI-EXPANDED, Science Citation Index Expanded; RCT, randomized controlled trial.

**Note1:**Publication-type restrictions for WoSCC and Scopus and language restrictions for all three databases were applied during screening rather than embedded in the search strings. The 2026 dataset was limited to records available through May 31, 2026. The three databases were screened and analyzed separately and were not merged into a single analytical dataset.The complete lists of the 250 included PubMed publications and the 11 excluded PubMed records are provided in Supplementary Tables S6 and S7, respectively.

**S3. Eligibility criteria and database-specific exclusions**

**Table S3. Eligibility criteria and reasons for exclusion**

| **Database/dataset** | **Inclusion criteria** | **Exclusion criteria** | **Screening outcome** |
| --- | --- | --- | --- |
| **Web of Science Core Collection (WoSCC)** Index: Science Citation Index Expanded (SCI-EXPANDED) | 1. Published from January 1, 2006 to May 31, 2026.  2. Published in English.  3. Document type classified as Article or Review Article.  4. Focused on music therapy or a broader music-based intervention in relation to cognitive impairment, mild cognitive impairment, dementia, Alzheimer's disease, cognitive decline, or cognitive dysfunction. | • Conference abstracts, editorial materials, book chapters, and other document types that were not Articles or Review Articles.  • Non-English publications.  • Records outside the prespecified publication period. | **Retrieved: 1,028**  **Excluded:**  • Non-eligible publication types: 167  • Non-English publications: 26  **Included: 835** |
| **Scopus** | 1. Published from January 1, 2006 to May 31, 2026.  2. Published in English.  3. Document type classified as Article or Review Article.  4. Focused on music therapy or a broader music-based intervention in relation to cognitive impairment, mild cognitive impairment, dementia, Alzheimer's disease, cognitive decline, or cognitive dysfunction. | •Conference abstracts, editorial materials, book chapters, and other document types not classified as Articles or Reviews.  • Non-English publications.  • Records outside the prespecified publication period. | **Retrieved: 1,831**  **Excluded:**  • Non-eligible publication types: 337  • Non-English publications: 126  **Included: 1,368** |
| **PubMed** Publication-level mapping of RCT-related research | 1. Published from January 1, 2006 to May 31, 2026.  2. Published in English.  3. Identified as a randomized controlled trial by publication type or explicitly described randomization in the title or abstract.  4. Included participants with cognitive impairment, mild cognitive impairment, dementia, Alzheimer's disease, or a closely related cognitive disorder.  5. Used music therapy, music listening, singing, therapeutic music, or another music-based approach as a central intervention.  6. Reported trial results suitable for publication-level descriptive and thematic mapping. | • Not an original eligible randomized-trial report after verification. • Study population did not meet the eligibility criteria. • Music was not used as the primary or central intervention. • Protocol, correction, intervention-development report, or other publication that did not report eligible trial results. • Secondary analysis of an existing randomized trial rather than a primary trial-results publication. | **Retrieved: 261** **Excluded:** • Protocol, correction, intervention-development, or other non-result publication: 4 • Ineligible study population: 4 • Not an original eligible randomized-trial report: 1 • Music not used as the primary intervention: 1 • Secondary analysis rather than a primary RCT report: 1 • **Total excluded: 11** **Included: 250** |

**Abbreviations:** WoSCC, Web of Science Core Collection; SCI-EXPANDED, Science Citation Index Expanded; RCT, randomized controlled trial.

**Note:** Exclusion reasons were assigned sequentially, and each record was counted once according to its primary reason for exclusion. The complete list of the 11 excluded PubMed records, including article titles, PMIDs or DOIs, exclusion categories, and record-specific reasons for exclusion, is provided in Supplementary Table S7. WoSCC, Scopus, and PubMed were screened and analyzed separately and were not merged into a single analytical dataset.

**Table S4. Literature screening and eligibility assessment procedures**

| **Component** | **Procedure** | **Database-specific application** |
| --- | --- | --- |
| **Record management** | Records retrieved from each database were organized and managed using R. R was also used to randomly select records for the post-screening agreement assessment. | WoSCC, Scopus, and PubMed |
| **Independent screening** | All retrieved records were screened independently and record by record by two reviewers, Qiangli Lei and Qing Liu. | WoSCC: 1,028 records; Scopus: 1,831 records; PubMed: 261 records |
| **Title and abstract screening** | Both reviewers independently assessed titles and abstracts against the predefined eligibility criteria. Full-text reports were consulted when the information in the title or abstract was insufficient to determine eligibility. | Applied to all three database-specific datasets |
| **WoSCC and Scopus assessment** | Records were assessed individually for topic relevance, eligible publication type, English language, and availability of the bibliographic information required for the planned analyses. | WoSCC and Scopus |
| **PubMed assessment** | All 261 PubMed records underwent title and abstract screening. Full texts were reviewed when eligibility could not be determined from the title and abstract. The final 11 excluded records were documented individually, with article titles, PMIDs or DOIs, and record-specific exclusion reasons provided in Supplementary Table S7. | PubMed |
| **Resolution of disagreements** | Disagreements between the two reviewers were resolved through discussion and consensus. No third reviewer was involved. | All three databases |
| **Agreement assessment** | After completion of screening, 50 records were randomly selected for a post-screening consistency assessment. Inter-reviewer agreement was evaluated using Cohen’s kappa coefficient. | Random sample of 50 screened records |
| **Agreement result** | The Cohen’s kappa coefficient was 0.85, indicating strong inter-reviewer agreement. | Post-screening consistency assessment |
| **Automation** | No automated screening, machine-learning, or artificial-intelligence tools were used to make eligibility decisions. | All three databases |
| **Documentation of exclusions** | The primary reason for exclusion was recorded for each excluded PubMed record. The exclusion categories comprised protocol, correction, intervention-development, or other non-result publication (n = 4); ineligible study population (n = 4); not an original eligible randomized-trial report (n = 1); music not used as the primary intervention (n = 1); and secondary analysis rather than a primary RCT report (n = 1). The complete record-level exclusion information is provided in Supplementary Table S7. | PubMed |
| **Database handling** | WoSCC, Scopus, and PubMed records were screened and analyzed separately. Records were not merged across databases, and no combined publication total was calculated. | All three databases |

**Abbreviations:** WoSCC, Web of Science Core Collection.

**Note:** The sample of 50 records was randomly selected after completion of the main screening process for an additional assessment of inter-reviewer agreement.

**Table S5. Duplicate checking and data-processing workflow**

| **Component** | **Procedure** | **Database-specific application** |
| --- | --- | --- |
| **Within-database duplicate checking** | Potential duplicate records were identified using the DOI as the primary matching field. For records without a DOI, the normalized article title, first author, publication year, and journal title were compared. All records flagged as potentially duplicated were manually verified before inclusion in the final database-specific datasets. | WoSCC, Scopus, and PubMed |
| **Cross-database handling** | Records retrieved from WoSCC, Scopus, and PubMed were not merged because the three databases served different analytical purposes. Publications appearing in more than one database were retained within their respective database-specific datasets, and no combined publication total was calculated. | All three databases |
| **Bibliographic data cleaning** | Bibliographic fields were checked and standardized before analysis. Obvious variants in author names, institutional names, country or regional names, journal titles, and keywords were reviewed and harmonized where appropriate. | Primarily WoSCC and Scopus |
| **Data verification** | Records with incomplete, inconsistent, or unclear bibliographic information were checked against the corresponding original database entries. Eligibility decisions and exclusion reasons were reviewed before the analytical datasets were finalized. | All three databases |
| **Record management** | Retrieved records, screening decisions, and exclusion reasons were organized and processed using R. The database-specific datasets were maintained separately throughout screening and analysis. | All three databases |
| **Analytical allocation** | The WoSCC dataset was used for the primary bibliometric and visualization analyses. Scopus was used as an independent dataset for cross-validation of key publication trends and geographic and institutional patterns. PubMed was used for publication-level descriptive and thematic mapping of randomized controlled trial-related research. | All three databases |
| **Final quality control** | Before analysis, the final datasets were checked for consistency between the reported retrieval counts, exclusion counts, and numbers of included records. Any discrepancy was resolved by rechecking the screening records and the original database entries. | All three databases |

**Abbreviations:** WoSCC, Web of Science Core Collection; DOI, digital object identifier; RCT, randomized controlled trial.

**Note:** Duplicate checking did not result in any additional within-database exclusions; therefore, no separate duplicate category was included in the screening counts.

## **Supplementary Table S6**

****Supplementary Table S6. Complete list of the 250 PubMed-indexed RCT-related publications included in the publication-level analysis****

To improve the transparency and reproducibility of the PubMed screening and publication-level analyses, the complete PubMed corpus is provided as separate supplementary tables. Supplementary Table S6 lists the 250 included PubMed-indexed RCT-related publications, including article titles, PMIDs, DOIs where available, authors, publication years, and journals. Supplementary Table S7 lists the 11 records excluded after eligibility assessment, together with their article titles, PMIDs or DOIs, exclusion categories, and record-specific reasons for exclusion.

## **Supplementary Table S7**

****Supplementary Table S7. Complete list of the 11 PubMed records excluded after eligibility assessment****

Supplementary Tables S6 and S7 are provided as separate Excel files to facilitate record-level verification and reuse.
